# Functional Outcomes of Clavicle Open Reduction and Internal Fixation (ORIF)

**DOI:** 10.7759/cureus.72048

**Published:** 2024-10-21

**Authors:** Muhammad Mannan, Usman Hafeez, Ahmed Hassan, Rimsha Tahir, Serajdin Ajnin

**Affiliations:** 1 Orthopedic Surgery, University Hospitals Birmingham NHS Foundation Trust, Birmingham, GBR; 2 Trauma and Orthopedics, University Hospitals Birmingham NHS Foundation Trust, Birmingham, GBR; 3 Orthopedics, University Hospitals Birmingham NHS Foundation Trust, Birmingham, GBR

**Keywords:** clavicle fracture, displaced midshaft fracture, functional outcome, orif, plating

## Abstract

Background: The clavicle is among the most commonly fractured bones. It can be managed nonoperatively or surgically. Open reduction and internal fixation (ORIF) surgery is the technique to help restore the clavicle’s anatomy and allow patients to rehabilitate sooner. The current study aims to assess the functional results and problems related to ORIF using plates for displaced midshaft clavicle fracture.

Methodology: This retrospective study was conducted in Heartlands Hospital, Birmingham, United Kingdom. A total of 60 patients who underwent ORIF for displaced midshaft clavicle fracture in Birmingham’s Heartlands Hospital from 2016 to 2023 were included in this study. Selected patients were reviewed to determine demographic data, cause of injury, fracture classification, and postoperative complications. All chosen patients were assessed for functional outcomes (Disabilities of the Arm, Shoulder, and Hand Questionnaire [DASH] score-based functional outcome measures).

Results: The mean age of patients was 34.75 ± 9.787 years (20-59 years). A total of 60 participants were included in the study, with 43 (71.7%) male and 17 (28.3%) female. In the majority of patients, the left side was affected (34 patients, 56.7%). The mode of injury was a fall from a bike or road traffic accident in 47 (78.3%) patients. The DASH score was excellent (98-100) in 49 (81.7%) patients, good to excellent (93-97) in 10 (16.7%) patients, and fair to good (82-92) in one (1.7%) patient. Of the total patients, 48.3% did not experience any complications. Among patients who developed complications, 20% of patients faced adhesive capsulitis or stiffness in the early postoperative period, which was treated with physiotherapy, followed by paresthesia over the surgical site, and anterior chest developed in 13.3%, followed by superficial infection in 3.3%.

Conclusions: ORIF for clavicle fractures ensures a high rate of bone union and improved functional outcomes after six months of surgery, with early pain relief and effective anatomical restoration. While it generally offers advantages over conservative methods, it is associated with a risk of adhesive capsulitis or stiffness.

## Introduction

The incidence of clavicle fractures ranges from 2.6% to 4% of all fractures, whereas shoulder girdle injuries account for 35% to 44% of cases. Clavicle fractures have a high incidence rate, including around 10% of all fractures in adults and 40% of injuries occurring in the vicinity of the shoulder girdle [1]. Around 80% of fractures manifest in the central region, where the application of typical compressive forces on the shoulder joint, along with the bone’s restricted cross-sectional area, leads to bone failure. Shaft fractures mostly affect individuals in their youth [2]. Clavicle fractures are frequent injuries in young, energetic people, particularly those who engage in hobbies or sports that involve high-speed falls (e.g., bicycling and motorbikes) or severe collisions (e.g., football and hockey) [3]. Clavicle fractures in adults may be treated conservatively or surgically. These fractures are mostly treated nonoperatively. Conservative management of a displaced fracture clavicle causes clavicle shortening, discomfort, strength loss, fast exhaustion, hyperesthesia of the hand and arm, difficulties sleeping on the affected side, and cosmetic issues. The high incidence of malunion and nonunion in displaced clavicle fractures treated conservatively led to the introduction of numerous forms of internal fixation procedures, which were also linked with varied comorbidities [4].

It has been shown that a surgical procedure for displaced midshaft clavicle fracture results in better union rates, greater early functional outcomes, and enhanced patient satisfaction [5]. The most frequent surgical operative therapy is ORIF, which uses wires, pins, or plates with screws [6]. Many implants are available for clavicle fixation, and plating is regarded as the gold standard. The clavicle’s distinctive anatomical form presents a problem for surgeons during reduction and implant insertion. The pre-contoured locking plate is currently the ideal implant for this kind of clavicle fracture because it gives improved stability and convenience of plate application [7]. This study aims to evaluate the functional outcomes and complications of open reduction and internal fixation (ORIF) using plates for displaced midshaft clavicle fractures, specifically assessing patient recovery using the Disabilities of the Arm, Shoulder, and Hand Questionnaire (DASH) score over a six-month follow-up period.

## Materials and methods

This retrospective study was carried out in Heartlands Hospital, Birmingham, United Kingdom. A total of 60 patients who underwent ORIF with plating for displaced midshaft clavicle fractures at Heartlands Hospital between 2016 and 2023 were included. Patients were aged 18 to 65 years and completed up to six months of follow-up after surgery. The exclusion criteria included an open fracture; a fracture that did not occur in the midshaft; a fracture that was pathological, delayed, or nonunion; and any associated vascular and neurological dysfunction. Medical files, charts, and radiographs were reviewed to determine patient demographics, cause of injury, fracture classification, implant selection, and postoperative complications. Patients were assessed for functional outcomes (DASH score). The goal of the surgery was to accomplish stable fixation of fractured ends while also restoring the clavicle’s length and curve, allowing for early shoulder mobilization. Following a pre-anesthetic examination, patients usually received surgery within two weeks of their injuries. Prophylactic antibiotics were administered before the incision. The procedure was performed under general anesthesia. A curvilinear incision was made above the clavicle to reveal the fracture. The fracture was reduced and held in place, with a plate inserted on the superior surface to use at least three screws in the primary proximal and distal pieces. Oblique fractures were repaired using a lag screw and neutralization plate. In transverse fractures, axial compression was produced, but in comminuted fractures, the bridge plate approach was employed. The deltopectoral fascia was closed as a separate layer. A collar and cuff sling was provided for two weeks. Sutures were removed on the 14th postoperative day.

## Results

The mean age of the patients was 34.75 ± 9.787 years (ranging from 20 to 59). Most patients (23, 38.3%) were 20 to 30 years old. The sample consisted of 60 patients, with 43 (71.7%) being male and 17 (26.3%) being female.

In the majority of patients, the left side was affected (34 patients, 56.7%). The mode of injury was a fall from a bike or road traffic accident in 47 (78.3%) patients, followed by a fall from stairs in eight (13.3%) patients. Robinson's classification showed that the type of fracture was 2B1 (simple or wedge comminuted) in four (81.7%) patients and 2B2 (isolated or comminuted segmental) in 11 (18.3%) patients. The injury-surgery interval was <7 days in 22 (36.67%) patients, 7-14 days in 34 (56.67%) patients, and >14 days in four (6.66%) patients. The demographic and clinical details of the patients are summarized in Table 1.

**Table 1:** Patient demographic and clinical details.

Variable	Frequency	Percentage (%)
Age groups		
20-30 years	23	38.3
31-40 years	21	35
41-50 years	11	18
>50 years	5	8.2
Gender		
Male	43	71.7
Female	17	28.3
Side		
Right side	26	43.3
Left side	34	56.7
Mode of injury		
Fall from height	47	78.3
Road traffic accident	8	13.3
Sports injuries	5	8.3
Robinson Classification		
2B1 (simple or wedge comminuted)	49	81.7
2B2 (isolated or comminuted segmental)	11	18.3
Injury–surgery interval		
<7 days	22	36.67
7-14 days	34	56.67
>14 days	4	6.66

The functional outcome of the patients was assessed on average using the DASH score. The DASH score was excellent (98-100) in 49 (81.7%) patients, good to excellent (93-97) in 10 (16.7%) patients, and fair to good (82-92) in one (1.7%) patient. The functional outcome of the patients is summarized in Table 2.

**Table 2:** Functional outcome of patients on DASH score. DASH, Disabilities of the Arm, Shoulder, and Hand Questionnaire

	Frequency	Percentage (%)
Excellent (98-100)	49	81.7
Good to excellent (93-97)	10	16.7
Fair to good (82-92)	1	1.7
Total	60	100

The results of the study showed that most (38, 63.3%) patients did not experience any complications. Among the patients who developed complications, 12 (20%) patients faced adhesive capsulitis or stiffness in the early postoperative period, which was treated with physiotherapy, followed by paresthesia over the surgical site, and anterior chest developed by 8 (13.3%), followed by superficial infection in two (3.33%). The complications experienced by the patients are detailed in Table 3.

**Table 3:** Complications of study patients (N = 60).

	Frequency	Percent
Superficial infection	02	3.33
Adhesive capsulitis	12	20.0
Paresthesia over the surgical site and anterior chest and anterior chest	8	13.3
None	38	63.3
Total	60	100

## Discussion

The ideal treatment for displaced midshaft clavicle fracture is still being debated. Conservative therapy for these fractures has a nonunion rate of roughly 5% [8]. For most clavicle fractures, nonoperative therapy remains the primary therapeutic option. Recent studies have shown a worse prognosis in instances with clavicle fractures treated nonoperatively in contrast with those treated surgically [9]. ORIF with plates is the primary surgical therapy for clavicular shaft fractures. Surgical therapy aims for anatomic reduction, rebuilding of clavicular length, and alignment of the shoulder girdle [10].

This retrospective study was carried out in Heartlands Hospital, Birmingham, United Kingdom. A total of 60 patients who underwent ORIF for displaced midshaft clavicle fracture in Heartlands Hospital from 2016 to 2023 were included in the study. The mean age of the patients was 34.75 ± 9.787 years, with an age range of 20 to 59 years. The sample consisted of 60 patients, with 43 (71.7%) being male and 17 (26.3%) being female. In the majority of patients, the left side was affected (34 patients, 56.7%). These results are in accordance with other studies. A study by Pillai [11] reported a sample with a mean age of 35 years and 70% male patients, which is similar to our study. In another study, there were 64% male patients [12].

In our study, the affected side was the left side in 34 (56.7%) patients, and the mode of injury was a fall in 47 (78.3%) patients, followed by a road traffic accident in eight (13.3%) patients, which is similar to samples used in other studies [7,13]. In one study, the mechanism of injury in 87% of patients was direct injury to the shoulder [11]. Robinson classification showed that type of fracture was 2B1 (simple or wedge comminuted) in 49 (81.7%) patients and 2B2 (isolated or comminuted segmental) in 11 (18.3%) patients. The injury-surgery interval was <7 days in 22 (36.67%) patients, 7-14 days in 34 (56.67%) patients, and >14 days in four (6.66%) patients. These findings are in accordance with other studies [14].

Surgical fixation of clavicular fracture is not without complication. The results of this study showed that most patients (38, 63.3%) did not experience any complications. Among patients who developed complications, 12 (20%) patients faced adhesive capsulitis or stiffness, followed by eight (13.3%) patients with paresthesia over the surgical site and anterior chest, followed by two patients (3.33%) with superficial infection. One study reported a complication rate of 33.33%. The most common complication was the reoperation rate, occurring in 30% of patients, followed by implant removal in 23.33%, implant failure in 6.66%, and superficial infection in 3.33% of cases [14]. Wani et al. documented that among its cases, the occurrence of problems included numbness in 12%, mechanical removal of the plate in 8%, nonunion in 4%, superficial wound infection in 4%, and symptomatic hardware in 4% [12].

The functional outcome of the patients was assessed on average using the DASH score. The DASH score was excellent (98-100) in 49 (81.7%) patients, good to excellent (93-97) in 10 (16.7%) patients, and fair to good (82-92) in one (1.7%) patient, which is comparable to other studies [15-17]. In another study, the average shoulder DASH score was 95.33 ± 3.4 in a one-year follow-up [18]. A study by Bhardwaj and Khader reported it was excellent [10]. The mean score was 91.86 after six months in a study by Ghosh et al. [7].

There are a few limitations to this study. This is a retrospective study with a small number of cases without having a control group (under conservative treatment). Therefore, a prospective study with a large population with a control group should be performed in the future to determine the clinical outcomes of ORIF in case of clavicle fracture.

## Conclusions

ORIF for clavicle fractures ensures a high rate of bone union and improved functional outcomes after six months of surgery, with early pain relief and effective anatomical restoration. While it generally offers advantages over conservative methods, it is associated with a risk of adhesive capsulitis or stiffness.
